# Exploiting *Leishmania*—Primed Dendritic Cells as Potential Immunomodulators of Canine Immune Response

**DOI:** 10.3390/cells13050445

**Published:** 2024-03-03

**Authors:** Ana Valério-Bolas, Mafalda Meunier, Joana Palma-Marques, Armanda Rodrigues, Ana Margarida Santos, Telmo Nunes, Rui Ferreira, Ana Armada, João Carlos Alves, Wilson Antunes, Inês Cardoso, Sofia Mesquita-Gabriel, Lis Lobo, Graça Alexandre-Pires, Luís Marques, Isabel Pereira da Fonseca, Gabriela Santos-Gomes

**Affiliations:** 1 Global Health and Tropical Medicine (GHTM), Associate Laboratory in Translation and Innovation towards Global Health, LA-REAL, Instituto de Higiene e Medicina Tropical (IHMT), Universidade NOVA de Lisboa (UNL), 1349-008 Lisbon, Portugal; ana.bolas@ihmt.unl.pt (A.V.-B.); a21001221@ihmt.unl.pt (M.M.); joanapmarques@ihmt.unl.pt (J.P.-M.); armanda.rodrigues@ihmt.unl.pt (A.R.); aarmada@ihmt.unl.pt (A.A.); a21000811@ihmt.unl.pt (S.M.-G.); lis.lobo@ihmt.unl.pt (L.L.); 2 Divisão de Medicina Veterinária, Guarda Nacional Republicana, 1200-771 Lisbon, Portugalalves.jca@gnr.pt (J.C.A.); 3 Microscopy Center, Faculty of Sciences, University of Lisbon, 1749-016 Lisbon, Portugal; telmonunes@hotmail.com; 4 Banco de Sangue Animal (BSA), 4100-462 Porto, Portugal; 5 Unidade Militar Laboratorial de Defesa Biológica e Química (UMLDBQ), 1849-012 Lisbon, Portugal; antunez.wdta@gmail.com; 6 CIISA, Centre for Interdisciplinary Research in Animal Health, Faculty of Veterinary Medicine, University of Lisbon, 1649-004 Lisbon, Portugal; gpires@fmv.ulisboa.pt (G.A.-P.); ifonseca@fmv.ulisboa.pt (I.P.d.F.); 7 Associate Laboratory for Animal and Veterinary Sciences (AL4AnimalS), 1200-771 Lisbon, Portugal; 8 BioSystems and Integrative Sciences Institute, Faculty of Sciences, University of Lisbon-FCUL-BioISI Ce3CE, 1749-016 Lisbon, Portugal

**Keywords:** dog, innate immunity, peripheral blood-derived dendritic cells (moDCs), *Leishmania amazonensis*, *L. infantum*, *Leishmania* extracellular vesicles, pattern recognition receptors, major histocompatibility complex, cytokines

## Abstract

Dendritic cells (DCs) capture pathogens and process antigens, playing a crucial role in activating naïve T cells, bridging the gap between innate and acquired immunity. However, little is known about DC activation when facing *Leishmania* parasites. Thus, this study investigates in vitro activity of canine peripheral blood-derived DCs (moDCs) exposed to *L. infantum* and *L. amazonensis* parasites and their extracellular vesicles (EVs). *L. infantum* increased toll-like receptor 4 gene expression in synergy with nuclear factor κB activation and the generation of pro-inflammatory cytokines. This parasite also induced the expression of class II molecules of major histocompatibility complex (MHC) and upregulated co-stimulatory molecule CD86, which, together with the release of chemokine CXCL16, can attract and help in T lymphocyte activation. In contrast, *L. amazonensis* induced moDCs to generate a mix of pro- and anti-inflammatory cytokines, indicating that this parasite can establish a different immune relationship with DCs. EVs promoted moDCs to express class I MHC associated with the upregulation of co-stimulatory molecules and the release of CXCL16, suggesting that EVs can modulate moDCs to attract cytotoxic CD8^+^ T cells. Thus, these parasites and their EVs can shape DC activation. A detailed understanding of DC activation may open new avenues for the development of advanced leishmaniasis control strategies.

## 1. Introduction

Leishmaniasis afflicts impoverished populations of subtropical and tropical countries and also affects animals, as is the case of domestic dogs. In the human population, *L. infantum* and *L. donovani* are responsible for human visceral leishmaniasis, and the Old World *L.major* and the New World *L. amazonensis, L. braziliensis,* and *L. mexicana* cause cutaneous leishmaniasis. *L. infantum* has been identified as the etiological agent of cutaneous leishmaniasis in humans. Canine leishmaniosis (CanL) is an endemic disease of global concern mainly caused by *Leishmania infantum.* However, in Central and South America, domestic dogs can be found infected by *L. amazonensis*, raising the hypothesis that the dog can also be a reservoir of New World species of *Leishmania* [1]. 

Innate immunity, the first line of defense against pathogens, includes phagocytes. Macrophages (MΦ) play a dual role by being professional antigen-presenting cells and the definitive *Leishmania*-host cell, and dendritic cells (DCs) can capture *Leishmania* parasites and process and present parasite antigens to lymphocytes [2]. DCs, first described by Steinman and Cohn in 1973 [3], are a heterogeneous cell population characterized by a star shape due to cytoplasmic extensions or dendrites. These cells are distributed in all peripheral tissues, as well as in primary (thymus and bone marrow) and secondary lymphoid (lymph nodes, payer plaques, and spleen) organs. DCs are derived from hematopoietic stem cells and can have two origins: (i) differentiated from myeloid progenitor cells expressing the CD11 protein (myeloid DCs, mDCs) and (ii) differentiated from lymphoid precursors expressing CD123 (plasmacytoid DC, pDCs).

Dendritic cells are professional antigen-presenting cells (APCs) that establish a bridge with T lymphocytes. By presenting pathogen antigens to lymphocytes, these cells provide signals that induce lymphocyte proliferation and activation. DCs are recognized as the most efficient cells in inducing the activation of T lymphocytes, being not only important in the activation of the acquired immune response, as they are crucial in the innate immune response [4]. In tissues and the bloodstream, mDCs and pDCs are in an immature state, being able to efficiently capture antigens at the sites of infection or inflammation. After recognition of the pathogen, these cells become activated and migrate to the secondary lymphoid organs, where they act as mature cells, presenting foreign antigens to T lymphocytes.

DCs interact with microorganisms through innate receptors, recognizing and capturing antigens associated with signaling molecules and target cell traffic. Antigen detection is mediated by different pattern recognition receptors (PRRs), the most prominent of which are Toll-like [5] and NOD-like receptors (NLRs). According to previous studies, NLRP10 mediates the migration of DCs into inflamed tissues, playing a crucial role at the beginning of the acquired immune response [6,7]. DCs do not possess conventional microbicidal mechanisms but present unique mechanisms for antigen processing and presentation via the major histocompatibility complex (MHC), since the proteolysis of antigens is directed to obtain smaller fragments and not to cause pathogen destruction [5].

Several reports have demonstrated a central role for DCs in orchestrating the immune response against *Leishmania* infection [8,9,10]. DCs can take up antigens via Fc receptors, C-type lectin receptors (CLRs), and PRRs. However, deficient expression of the TLR adaptor MyD88 results in impaired DC activation and lower levels of IL-12 production, both essential elements in the assembly of protective immunity against *Leishmania*. In addition, in vivo studies showed that neutralization of TLR2 and TLR4 reduced the expression of co-stimulatory molecules in *L. major*-infected DCs [11]. The absence of TLR2 in *L. braziliensis*-infected mice enhanced DC activity and increased IL-12 production. Thus, TLR2^−/−^ DCs infected with *L. braziliensis* were more competent at priming naïve CD4^+^ T cells, which was associated with increased IFN-γ production and greater resistance to infection [12]. *L. major* appears to signalize through TLR2, TLR4, and TLR9 [13,14,15,16,17]. In vivo assays with *L. major* infection also confirmed the importance of TLR9 in IL-12 production by DCs [15,16].

Antigen presentation and IL-12 production by DCs are critical for the differentiation of CD4^+^ Th1 and CD8^+^ T cells to mediate a protective immune response against *Leishmania* infection [18,19]. DCs appear to be the main source of IL-12 in early *Leishmania* infection. Thus, DCs derived from the skin of C57BL/6 mice and splenic DCs located in the periarteriolar lymphoid sheath of BALB/c mice were recognized as the main source of IL-12p40 immediately after dermotropic infection by *L. major* or viscerotropic infection by *L. donovani*, respectively [8,20]. The effect of *Leishmania* infection on IL-12 induction and DC maturation may vary between DC subtypes and *Leishmania* species. Indeed, in vitro infection of murine bone marrow-derived DC with *L. mexicana* promastigotes failed not only to induce IL-12 release but also to activate immature DCs [21]. Subsets of murine splenic DCs may differ in their ability to produce IL-12 and phagocytose *L. major* amastigotes. According to the studies reported by Quinones and colleagues [20], human and murine DCs appear to have a pre-formed membrane-associated pool of IL-12p70 that is rapidly released upon contact with *L. donovani*.

Therefore, a considerable number of studies in humans and mice have demonstrated the role of DCs in orchestrating the immune response against *Leishmania* infection. However, little information is available on the immune functionality of canine DCs when facing *Leishmania* parasites.

*Leishmania* parasites have evolved different strategies to escape the host’s immune response, including the use of extracellular vesicles (EVs) to establish communication with the host immune cell. EVs shed by *Leishmania* parasites are nanosized lipid vesicles carrying parasite-derived macromolecules. Up to 329 molecules were identified in EVs released from axenic promastigotes, of which 52% are representatives of the parasite’s secretome. It has been previously reported that EVs of *Leishmania* spp. contain virulence factors, such as glycoprotein of 63kDa (gp63), heat shock protein (HSP) 10, HSP70, 14-3-3-like protein, lipophosphoglycan (LPG), and elongation factor 1 (EF-1) [22,23,24,25]. Through EV shedding, *Leishmania* parasites can interfere with both innate and adaptive host immunity [22], indicating that EVs play a role in parasite infection and the possibility that the early innate immune response against *Leishmania* parasites, including DC activation, is influenced by EVs.

Thus, the present study examines the modulation of canine DC immune activation by *L. infantum* and *L. amazonensis* parasites and by EVs shed by both parasites and how they can influence the establishment of the bridge between innate and adaptive immunity. To this end, an in vitro model based on canine DCs was established, and peripheral blood monocyte-derived DCs (moDCs) were used to assess PRR transcription and nuclear factor κB (NF-κB) activation, in addition to MHC surface expression, cytokine and co-stimulatory molecule gene expression, and chemokine production.

## 2. Materials and Methods

### 2.1. Peripheral Blood Monocyte-Derived Dendritic Cells

Peripheral blood from clinically and analytically healthy dogs was used to differentiate DCs (moDCs). Dogs also tested negative for anti-*Leishmania* antibodies (Kit Anti-*Leishmania* Antibodies, BioSystems S.A, Barcelona, Spain) and parasite DNA by qPCR [26]. The study was carried out in conformity with the institutional guidelines and the EU requirements and was approved by the Ethics and Welfare Committee of the Faculty of Veterinary Medicine, University of Lisbon (Ref. 008/19).

Canine peripheral blood was collected into a tube with the anticoagulant citrate phosphate dextrose adenine solution (CPDA), and blood monocytes were isolated according to Pereira and colleagues [27]. Briefly, peripheral blood was overlaid on a gradient of Hystopaque^®^ density 1077 (Sigma-Aldrich, St. Louis, MA, USA) and centrifuged. The interface cell ring, enriched in monocytes, was collected and washed. The remaining red blood cells were lysed, and the concentration of viable monocytes was determined by trypan blue exclusion in a Neubauer chamber under an optical microscope (Motic B1-220E-SP, Xiamen, China). Monocyte differentiation into moDCs was induced by incubating monocytes in Roswell Park Memorial Institute medium 1640 (RPMI, Sigma-Aldrich) supplemented with 10% (*v*/*v*) of heat-inactivated fetal bovine serum (hiFBS, Sigma-Aldrich), 10% (*v*/*v*) of colony-stimulating factor (CSF), and 100 ng·mL^−1^ of canine recombinant IL-4 (rcaIL-4, R&D Systems, Minneapolis, MN, USA) for seven days at 37 °C in a humidified atmosphere containing 5% CO_2_ [28,29].

### 2.2. moDC Immunophenotyping

To establish the molecular signature, moDC was immunophenotyped by multiparametric flow cytometry analysis for classical surface biomarkers (CD1a, CD11c, CD14, and CD83).

Cells were washed with cold 1× phosphate-buffered saline (PBS) (Lonza, Verviers, Belgium) (300× *g*, 10 min, 4 °C) and incubated for 30 min at 4 °C in PBS 2% (*v*/*v*) FBS with the following monoclonal antibody-directed conjugates: ALEXA Fluor 647-conjugated mouse anti-human CD1a (clone NA1/34-HLK, Bio-Rad, Hercules, CA, USA), APC-conjugated mouse anti-human CD11c (clone Bu15, Thermo Fisher Scientific, Waltham, MA, USA), FICT-conjugated mouse anti-human CD14 (clone TuK4, Thermo Fisher Scientific), and PE-conjugated mouse anti-human CD83 (clone HB15e, Invitrogen, Waltham, MA, USA) (Supplementary Table S1). Cells were then fixed with 2% (*m*/*v*) paraformaldehyde (Sigma-Aldrich) in PBS and washed. Cell acquisition was performed on a CytoFLEX system cell analyzer (Beckman Coulter, Brea, CA, USA), and data were analyzed by CytExpert v2.5 software (Beckman Coulter, USA). A singlet gate was used to define the non-clumping cells based on pulse geometry FSC-H vs. FSC-A.

### 2.3. Leishmania Parasites

*L. infantum* and *L. amazonensis* promastigotes were maintained at 24 °C in Schneider Drosophila medium with L-glutamine (SCHN, Sigma-Aldrich) supplemented with 10% (*v*/*v*) hiFBS and penicillin–streptomycin (Biochrom) at 100 U/mL and 100 μg·mL^−1^ (SCHN medium). Promastigotes were used to infect moDCs.

Green fluorescent protein (GFP)-expressing *L. infantum* promastigotes [30] were grown in SCHN medium supplemented with 25 μg·mL^−1^ of geneticin (Sigma-Aldrich). Fluorescent promastigotes were confirmed under a fluorescent microscope equipped with a GFP filter (Olympus 5X, Tokyo, Japan). Cultured GFP-promastigotes were used to infect moDCs.

### 2.4. Purification of Extracellular Vesicles Shed by L. infantum and L. amazonensis-Cultured Promastigotes

**Figure 1:**
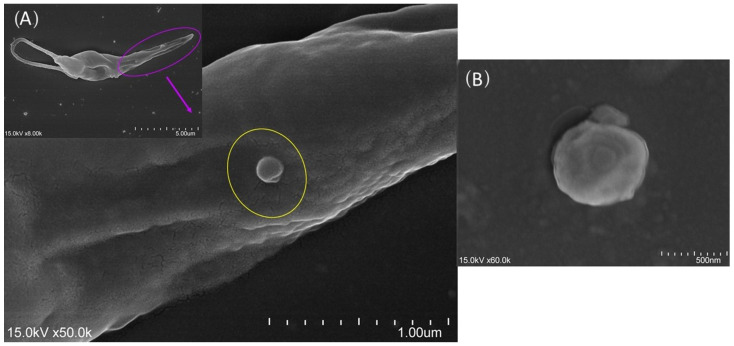
Extracellular vesicles (EVs) budding from *Leishmania* promastigotes. The cultured *Leishmania infantum* promastigote was observed by scanning electron microscope, and images were acquired. An extracellular vesicle (**A**) surrounded by a yellow circle and a free EV (**B**) can be observed.

To obtain purified EVs shed by *L. infantum* (LiEVs) and *L. amazonensis* (LaEVs) (Figure 1) promastigotes were incubated for 72 h in SCHN medium supplemented with 10% (*v*/*v*) exosome-depleted FBS (exo-free FBS, Gibco™, Thermo Fisher Scientific, Waltham, MA, USA), as described in Weber and coworkers [31]. Briefly, cultures were centrifuged at 3000× *g* for 30 min at 4 °C to remove dead parasites and debris. The supernatant was removed, and the total exosome isolation reagent (Invitrogen™, Thermo Fisher Scientific) was added at an isolation reagent–supernatant ratio of 1:2. The suspension was incubated overnight at 4 °C, then centrifuged at 10,000× *g* for 1 h at 4 °C. The pellet was resuspended in a convenient volume of 1× PBS, and the protein concentration was assessed using a spectrophotometer (NanoDrop 1000, Thermo Fisher Scientific). Suspensions were conserved at −80 °C until further use.

### 2.5. moDC Activation

Dog moDCs (1 × 10^5^ cells/well) were seeded in 96-well plates, and *L. infantum* and *L. amazonensis* promastigotes were added at a parasite–moDC ratio of 5:1 in 300 µL of RPMI supplemented with 10% hiFBS (*v*/*v*). moDCs were also stimulated by *L. infantum* (LiEVs) and *L. amazonensis* (LaEVs) EVs (10 μg·mL^−1^) in RPMI supplemented with exo-free FBS. Plates were incubated for 24 h at 37 °C in a humidified atmosphere containing 5% CO_2_. In parallel, a resting moDC was used as a non-activated control. After incubation, supernatants were used to determine CXCL16 production; cells were utilized for MHC detection and to quantify the gene expression of PRR, co-stimulatory molecules, and cytokines; and cell lysates were employed to examine the activity of nuclear factor-κB (NF-κB).

### 2.6. Microscopic Images of moDCs

To examine the morphology of moDCs and the interaction of these cells with *Leishmania* parasites, light microscopy was used, as well as fluorescent, confocal, and scanning electron microscopy (SEM).

Cultured peripheral blood monocytes were regularly observed by inverted light microscopy (Olympus, Japan) until morphology was consistent with DCs. Then, moDC cytospin slides were exposed to parasites for 24 h and examined under a light microscope after being stained with Giemsa dye.

moDCs were cultured on round coverslips in 24-well plates at 37 °C in a humidified atmosphere of 5% CO_2_. *L. infantum* GFP-promastigotes were added to the cultured cells at a 3:1 ratio. After 5 h of incubation, coverslip-adherent cells were washed with 1× PBS and fixed with PBS 2% paraformaldehyde for 20 min on ice. After fixation, the coverslips were incubated with Dil Stain (1,1’-dioctadecyl-3,3,3’,3’-tetramethylindocarbocyanine perchlorate (DiI, Invitrogen™ D282)) for 2 h at room temperature, then washed two times with PBS. DiI is a lipophilic fluorescent dye that stains the cell membrane orange/red and diffuses laterally, staining the entire cell. It is a weakly fluorescent compound until it is incorporated into cell membranes. The cover slides were observed by confocal microscopy (Leica SPE running LAS X 3.5.7.23225).

For cell examination under fluorescent microscopy, cells were stained with DAPI (4′,6-diamidino-2-phenylindole, VWR). DAPI is a blue fluorescent dye that binds to AT-rich regions of double-strand DNA, showing about a 20-fold increase in fluorescence intensity. Since DAPI crosses intact cell membranes, it can be used as a nuclear counterstain. 

To observe cell topography, moDCs were incubated with *L. infantum* and *L. amazonensis* parasites in the proportion of 1 moDC: 3 promastigotes for 1.5 h and 5 h at 37 °C in a humid atmosphere of 5% CO_2_. After the incubation, cells were fixed on coverslips with 2.5% glutaraldehyde and 0.1 M sodium cacodylate buffer (pH 7.4) for 2 h at 4 °C. Afterward, coverslips were washed, then dehydrated by sequential addition of 30%, 50%, 70%, 80%, and 90% ethanol for 5 min each and immersed in 100% ethanol. Then, cells were treated with hexamethyldisilazane solvent (Sigma-Aldrich), coated with gold–palladium (91017-AU), and mounted on stubs to be observed under an ultra-high-resolution scanning electron microscope (SU8010 Hitachi, Tokyo, Japan). After microscopic examination, digital images were acquired.

### 2.7. moDC Viability

To ensure that moDCs were still viable after parasite infection and EV stimulation, pre-apoptotic, apoptotic, and viable cells were assessed by multiparametric flow cytometry analysis using a commercial TACS^TM^ Annexin V FITC kit (R&D Systems, Minneapolis, MN, USA), according to the manufacturer’s instructions.

Cells were washed with 200 mL of cold 1× PBS (300× *g*, 10 min, 4 °C) and incubated with the a commercial TACS^TM^ Annexin V FITC kit (R&D Systems) according to the manufacturer’s instructions. FL1-H (Annexin V FITC) vs. FL5-H (PI) gate on an untreated moDC was used to delimit the annexin V FITC^−^/PI^−^ population (viable cells), annexin V FITC^+^/PI^−^ (pre-apoptotic cells), and annexin V FITC^+ or −^/PI^+^ cells (apoptotic cells).

### 2.8. Gene Expression of PRRs, Cytokines, and Co-Stimulatory Molecules

The immune activation of moDCs generated against *L. infantum* and *L. amazonensis* parasites and induced by EVs (LiEVs and LaEVs) were examined through the gene expression of cytokines (*IL-1β, IL-8, IL-10, IL-12p35, IL-12p40, IL-18,* and *TGF-β*), intramembrane (*TLR2*, *TLR4*, and *TLR9*) and cytoplasmatic (*NOD1*, *NOD2*, and NLRP10) PRRs, and the *CD80* and *CD86* co-stimulatory molecules by real-time PCR.

RNA extraction of moDCs was performed using an NZY Total RNA Isolation kit (NZYTech—Genes & Enzymes) according to the manufacturer’s instructions, followed by digestion of contaminant DNA with DNAse to maximize the RNA purity. The purity of isolated RNA was confirmed by a Nanodrop^®^ 1000 spectrophotometer at an absorbance ratio of 260/280 nm. RNA samples with an absorbance ≈2 were used, which indicates that the samples were constituted by pure RNA.

RNA was transcribed into complementary DNA (cDNA) using an NZY First-strand cDNA Synthesis Kit (Nzytech-Genes & Enzymes, Lisbon, Portugal) according to the manufacturer’s instructions. cDNA strands were then used as a template for posterior amplification. Degradation of the cDNA:RNA hybrids formed after the first-stranded cDNA synthesis was ensured by the addition of RNase H.

Amplification was carried out as described by Rodrigues and colleagues [32]. Primers (Supplementary Table S2) were either selected from published works [32,33,34,35,36,37,38,39,40] in an extended literature review or designed using Primer3 software 4.1.0 [41,42]. For the absolute quantification of DNA copies, external cDNA standards were constructed for all target genes by cloning PCR fragments generated by the same primers into a pGEM^®^-TEasy Vector according to the manufacturer’s recommendations (Promega, Madison, WI, USA) as previously described by Rodrigues and coworkers [43], and calibration curves were established for each gene.

To correct possible flaws in the reverse-transcription reaction, β-actin was used as an endogenous control. The PCR reaction mixture was prepared with cDNA, SsoAdvanced Univ SYBR Green Supermix (Bio-Rad), forward and reverse primers, and ultra-pure water. Amplification was performed in a Bio-Rad CFX Maestro PCR System thermal cycler (Bio-Rad, UK) under the following conditions: 5 min at 95 °C for complete DNA denaturation, 40 cycles of 30 s at 95 °C and 30 s at primer/gene-specific temperature for annealing and extension, and 90 cycles of 10 s at a starting temperature of 50 °C with an increment of 0.5 °C for each cycle. The fluorescence levels of each sample were analyzed in real time by the thermal cycle, and the amount of amplified DNA was calculated by comparison with the calibration curves. For each gene, the results are expressed as the number of gene copies per 1000 copies of β-actin.

### 2.9. NF-κB Activation

To investigate the activation of sensing pathways after recognition of parasite-common antigenic patterns, the expression and translocation of NF-κB [44] were estimated by commercial colorimetric immunoassay.

Lysates of moDCs previously incubated for 24 h with *L. infantum* or *L. amazonensis* promastigotes or EVs (LiEVs and LaEVs) and resting moDCs were used to quantify NF-κB activation.

A Canine Nuclear Factor Kappa B (NF-κB) ELISA kit (BlueGene Biotech CO., LTD, China) was used to estimate NF-κB according to the manufacturer’s instructions. The resulting color was measured at 450 nm using a plate reader (TRIADTM 1065, DYNEX Technologies, Chantilly, VA, USA), and the concentration of NF-κB in each sample was calculated by regression analysis.

### 2.10. Chemokines

The ability of moDCs to recruit other immune cells was evaluated through the production of CXCL16 and IL-8 gene expression.

CXCL16 concentration was estimated in the supernatants of moDCs incubated with *L. infantum* and *L. amazonensis* promastigotes, EV (LiEVs and LaEVs)-stimulated moDCs, and unpulsed moDCs by a commercial competitive ELISA (Canine CXC Chemokine Ligand 16 (CXCL16) ELISA Kit, MyBioSource, San Diego, CA, USA) following the manufacturer’s instructions. The absorbance was read at 450 nm through a plate reader. To estimate the CXCL16 concentration in the moDC samples, a standard curve was constructed using 100 µL of standards with known concentrations of chemokine ranging between 0 pg and 1000 pg, and the cytokine concentration was calculated by regression analysis.

### 2.11. Surface Expression of MHC Molecules

The expression of MHC class I (MHCI) and MHC class II (MHCII) molecules at the surface of unpulsed moDCs was examined by multiparametric flow cytometry. *L. infantum*- and *L. amazonensis*-exposed moDCs, moDCs stimulated by EVs (LiEVs and LaEVs), and unpulsed moDCs were washed with 1× PBS at 500× *g* for 10 min. Cells were then fixed with 2% paraformaldehyde for 20 min at room temperature and incubated with the rat anti-dog MHCI (W6/32, Bio-Rad) and mouse anti-human MHCII (clone YKIX334.2, Bio-Rad) monoclonal antibodies directly conjugated with fluorescent dyes (Supplementary Table S1). After incubation, cells were washed with 1× PBS (500× *g* for 10 min) and acquired by a flow cytometer analyzer, and the median fluorescence intensity (MFI) was analyzed. FSC-H vs. FSC-A was employed to define a singlet gate of non-clumping cells.

### 2.12. Data Analysis

Assays were realized in blood samples of at least six dogs, except microscopy, which was performed with samples of three dogs. The non-parametric Wilcoxon test for paired samples was used to perform the statistical analysis. Differences were considered significant with a 5% significance level (*p* < 0.05). GraphPad Prism version 9 for Windows (GraphPad Software, La Jolla, CA, USA) was used for statistical analysis, and data were graphically represented, taking into consideration possible outliers, since the blood samples used in this study come from a natural population of dogs, and no other restrictions were imposed besides being clinically healthy and negative for antileishmanial antibodies.

## 3. Results

### 3.1. Monocyte-Differentiated Dendritic Cells Exhibit Characteristic Morphology

**Figure 2:**
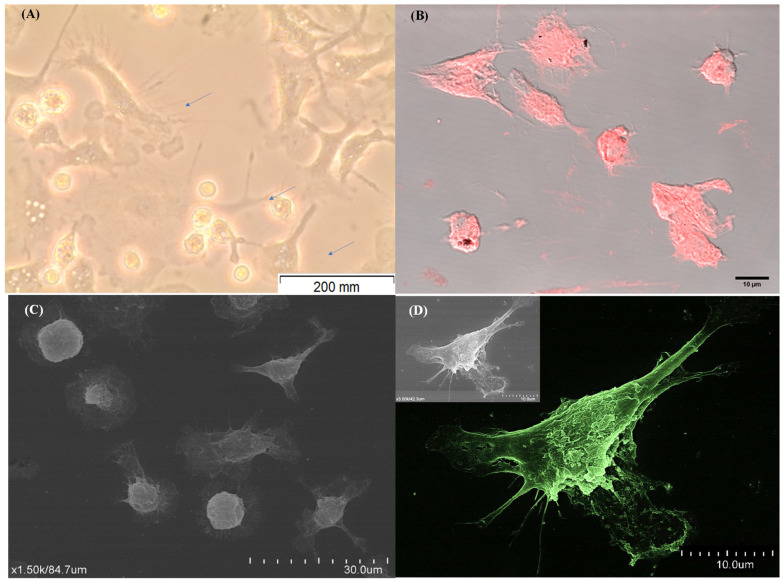
Morphological differentiation of monocyte-derived dendritic cells. After 7 days of differentiation, the in vitro morphological changes of mononuclear cells were observed by inverted light microscopy ((**A**), ×40 magnification) and by confocal microscopy (**B**), and digital images were acquired. Blue arrows indicate cell cytoplasmic projections. Cell topography (**C**,**D**) was observed by scanning electron microscopy at different magnifications. Image D was artificially colored using GIMP2.10.32 software.

The methodology implemented to achieve mature DCs derived from blood monocytes (moDCs) showed that the cells exhibited a uniform round shape after 24 h and 72 h of incubation. However, after 7 days of differentiation, there was a remarkable change in cell morphology. Cells showed an elongated shape and irregular surface and displayed filiform cytoplasmic projections consistent with the conventional DC morphology (Figure 2A,B). Moreover, it is also possible to verify that moDCs showed a distinct topography, exhibiting a star shape with cytoplasmatic projections (dendrites) and a less irregular surface (Figure 2C,D).

Thus, the implemented in vitro methodology directs the differentiation of cells with morphology consistent with DCs, and 7 days of differentiation seems to be the optimal time to obtain canine moDCs.

### 3.2. Monocyte-Differentiated Cells Exhibit a Molecular Signature Compatible with DCs

**Figure 3:**
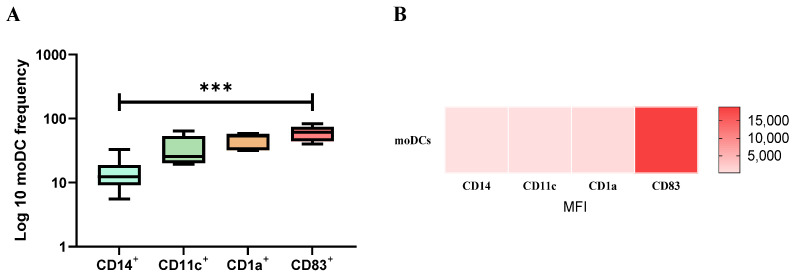
Phenotypic profile of moDCs. moDCs were labeled with directly conjugated anti-CD1a, CD11c, CD14, and CD83 monoclonal antibodies, and the frequency of moDC (**A**) subsets and the mean fluorescence intensity (MFI) of each fluorometer (**B**) were evaluated by multiparametric flow cytometry. The results of cell frequency of at least six blood samples determined in duplicate are represented by box plots and whiskers (minimum to maximum), and MFI data are represented by a heat map. The non-parametric Wilcoxon test was used for statistical comparisons. *** (*p* < 0.001) indicates statistically significant differences.

Most moDCs exhibited a CD11c^+^, CD1a^+^, and CD83^+^ phenotype (Figure 3A), whereas a lower frequency of cells expressed CD14. Transmembrane protein CD1a mediates the presentation of lipid antigens, integrin CD11 is fundamental in the cell adhesion process, and the CD83 molecule determines the maturation state of DCs. Moreover, the frequency of cells that express CD14 and CD83 molecules were significantly different in moDCs (*p* = 0.0005). Moreover, moDCs presents higher surface expression levels of CD83 (Figure 3B).

Therefore, after the in vitro differentiation process, most moDCs present surface markers of DCs and high expression of CD83, which points toward the differentiation of mature dendritic cells.

### 3.3. moDCs Bind and Internalize L. infantum and L. amazonensis Parasites

**Figure 4:**
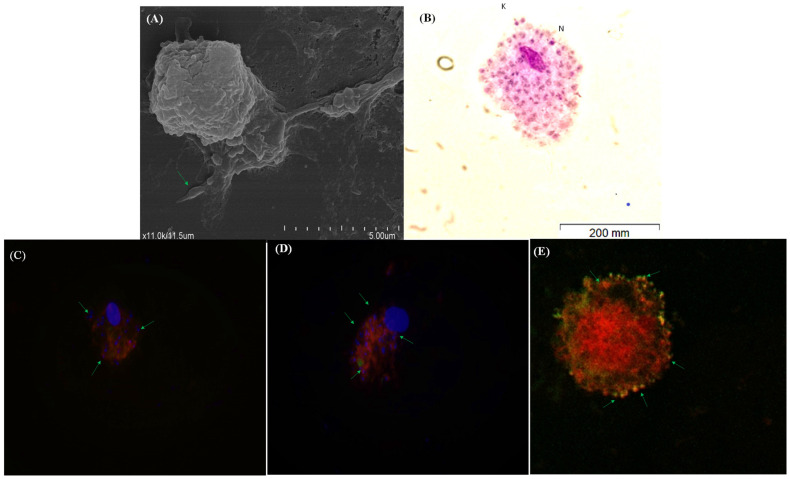
*L. infantum* and *L. amazonensis* parasites bind to moDCs and are uptaken. The interaction of promastigotes with moDC was observed by SEM (**A**). Arrows point to promastigotes. moDCs incubated with *L. amazonensis* promastigotes for 24 h (**B**) were observed by optical microscopy (size bar—200 nm, ×1000 magnification). moDCs incubated with *L. infantum* GFP promastigotes for 24 h were observed by fluorescence (×1000 magnification) (**C**,**D**) and confocal microscopy (**E**). Red—cytoplasm (**C**–**E**); blue—the nuclei of moDCs and *Leishmania* amastigote forms (**C**,**D**); yellow/orange color—amastigote forms (**E**); green arrows indicate parasites; n—amastigote nucleus; K—amastigote kinetoplast.

To examine if moDCs are functional and can recognize and internalize pathogenic agents, the interaction of moDCs with *Leishmania* parasites was observed by SEM. After 5 h of incubation (Figure 4A), promastigotes seemed to establish contact with dendrites. moDCs exposed to *L. amazonensis* promastigotes observed by optical microscopy (Figure 4B) and *L. infantum* GFP promastigotes analyzed by fluorescence (Figure 4C,D) and confocal (Figure 4E) microscopy show intracellular amastigote forms. Thus, moDCs recognize *Leishmania* promastigotes of two different species, internalize the parasites, and support differentiation into amastigotes.

**Figure 5:**
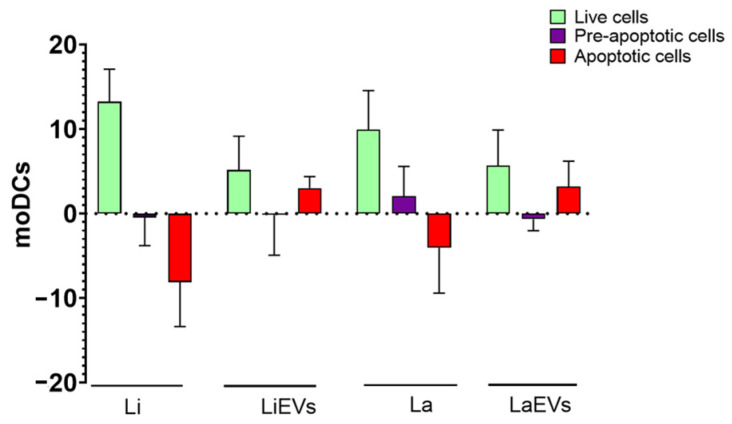
Viability of *L. infantum*- and *L. amazonensis*-infected moDCs. moDCs infected by *L. infantum* (Li) or *L. amazonensis* (La) promastigotes or stimulated by *L. infantum* (LiEVs) or *L. amazonensis* (LaEVs) EVs for 24 h were assessed by multiparametric flow cytometry, using annexin-V and propidium iodide. The mean and standard deviation of live cells, pre-apoptotic cells, and apoptotic cells of six samples normalized to unpulsed moDCs are represented by a column bar graph.

The effect of internalized parasites and EVs on moDC viability was also evaluated by multiparametric flow cytometry. Compared to non-activated moDCs, *L. infantum*- or *L. amazonensis*-infected moDCs showed a significant increase in viable cells (*p* = 0.0313) and a decrease in apoptotic cells. In turn, exposure to parasite EVs promoted a slight increase in viable and apoptotic moDCs (Figure 5).

Thus, moDCs recognize *Leishmania* promastigotes of two different species, internalize the parasites, and support differentiation into amastigotes. *Leishmania* infection appears to favor the life span of moDCs, whereas EVs do not appear to be a major cause of cell damage.

### 3.4. L. infantum Parasites Promote TLR4 Gene Expression and Activate NF-κB

Innate immune receptors play a crucial role in recognizing pathogen molecular patterns. Thus, to investigate the effect of parasites and EVs in PRRs of moDCs, the gene expression of cell membrane (*TLR2* and *TLR4*), endocytic (*TLR9*), and cytoplasmatic (*NOD1*, *NOD2*, and *NLRP10*) innate receptors was estimated by reverse transcriptase quantitative PCR in *L. infantum*- and *L. amazonensis*-infected moDCs, moDCs exposed to parasite EVs (LiEVs and LaEVs), and unpulsed moDCs.

**Figure 6:**
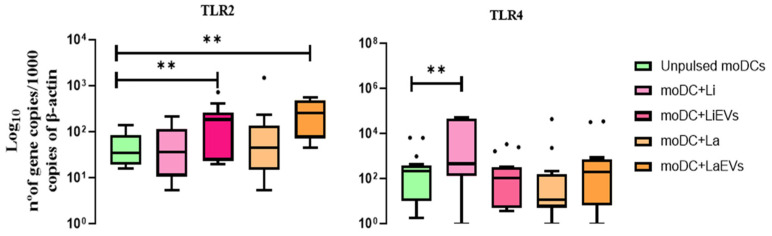
Gene expression of *TLR2* and *TLR4* by *L. infantum*- and *L. amazonensis*-infected moDCs. RNA extracted from infected moDCs (moDC + Li and moDC+La) and moDCs exposed to parasite EVs (moDCs + LiEVs and moDCs + LaEVs) for 24 h was used to evaluate *TLR2* and *TLR4* gene expression. Results of at least 10 samples are represented by box plots, including the median, interquartile ranges, and minimum and maximum values. Black dots are indicative of outliers. Nonparametric Wilcoxon’s test was used for statistical comparisons. ** (*p* < 0.001) indicates statistically significant differences.

In comparison with unpulsed moDCs, *TLR2* gene expression registered a significant increase in moDCs exposed to EVs (*p*_LaEVs_ = 0.0020, *p*_LiEVs_ = 0.0098) (Figure 6), while *L. infantum*-infected moDCs showed *TLR4* upregulation (*p* = 0.0035).

In contrast, *NOD1* gene expression significantly decreased in moDCs exposed to LiEVs (*p* = 0.0385) when compared to unpulsed moDCs. *L. infantum*-infected moDCs showed a marked downregulation (*p* = 0.0068) of *NLRP10* gene expression, and the accumulation of TLR9 mRNA significantly decreased in *L. amazonensis*-infected moDCs (*p* = 0.0052) and in moDCs exposed to *L. infantum* EVs (*p* = 0.0034). In turn, *NOD2* does not show significant differences regarding infected moDCs or EV-stimulated moDCs (Supplementary Figure S1).

**Figure 7:**
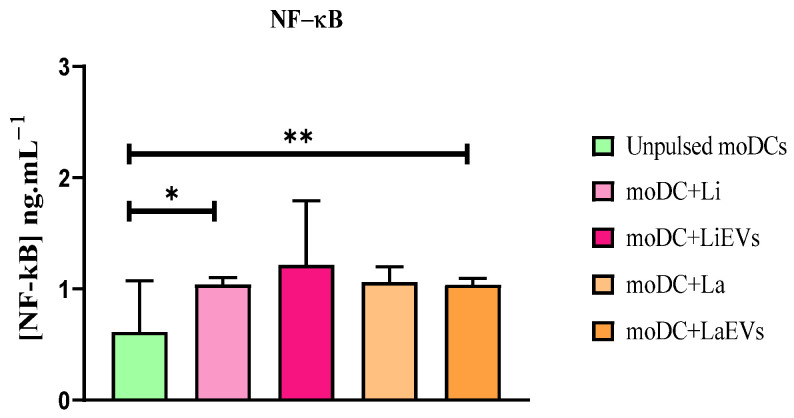
Activation and translocation of NF-κB to the moDC nucleus. Lysates of infected moDCs (moDCs + Li and moDCs + La) and moDCs exposed to parasite EVs (moDCs + LiEVs and moDCs + LaEVs) for 24 h were analyzed by ELISA. In parallel, unpulsed moDCs were also evaluated. The mean and standard deviation of eight samples are represented by a column bar graph. Wilcoxon’s nonparametric test was used for statistical comparisons. * (*p* < 0.05) and ** (*p* < 0.01) indicate significant differences.

After recognition of parasite antigens, signalization of PRR downstream pathways can lead to activation and translocation of NF-κB to the nucleus, which promotes the transcriptional activity of pro-inflammatory mediators, including chemokines and cytokines. *L. infantum*-infected moDCs (*p* = 0.0234) and moDCs exposed to LaEVs (*p* = 0.0078) exhibited a significantly high expression and translocation of NF-κB to the nucleus when compared to unpulsed moDCs (Figure 7).

Thus, *L. infantum* and *L. amazonensis* parasites show different effects on gene expression of moDC sensors, as well as on NF-κB activation and translocation. *L. amazonensis* appeared to downregulate *TLR9* expression, and *L. infantum* inhibited the accumulation of NLRP10 mRNA. However, EVs triggered the expression of *TLR2* and *TLR4*. Despite moDCs expressing surface-sensing genes, only LaEVs and *L. infantum* parasites seemed to induce NF-κB activation. On the other hand, *L. amazonensis* infection does not seem to modulate the gene expression of PRRs evaluated in the current study.

### 3.5. L. infantum and L. amazonensis EVs Promote Surface Expression of MHCI and Upregulate CD80 and CD86 Co-Stimulatory Molecules

MHC molecules expressed by DCs have a central role in guiding the T-cell immune response, establishing a bridge between innate and adaptive immunity. When complexed with parasite antigens, surface MHCI molecules promote the activation of cytotoxic T cells, and MHCII molecules induce the activation of T-helper cells. Thus, to indirectly evaluate the ability of *L.infantum*- and *L. amazonensis*-infected moDCs and moDCs exposed to EVs (LiEVs and LaEVs) presenting antigens to lymphocytes, the surface expression of MHCI and MHCII was evaluated, as well as the gene expression of co-stimulatory molecules *CD80* and *CD86*. These co-stimulatory molecules expressed in the cellular membrane of DCs play a crucial role in ensuring the effective start of T cells’ immune response.

**Figure 8:**
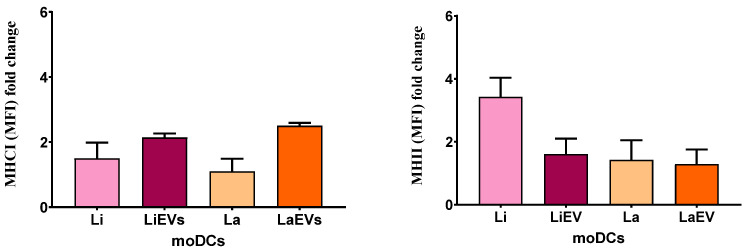
Expression levels of MHC molecules in *L. infantum*- and *L. amazonensis*-infected moDCs. The molecular fluorescent intensity (MFI) of MHCI and MHCII molecules was evaluated by multiparametric flow cytometry in infected moDCs (LiEVs and LaEVs) and moDCs exposed to parasite EVs (LiEVs and LaEVs). The fold change of six samples compared with unpulsed moDCs is represented by column bars (mean and SEM).

Primed moDCs showed an increased trend towards MHC expression. EVs of both *Leishmania* species caused a two-fold increase in MHCI expression in moDCs (Figure 8) and *L. infantum*-infected moDCs showed at least a three-fold increase in the expression of MHCII. when compared with non-activated moDCs.

**Figure 9:**
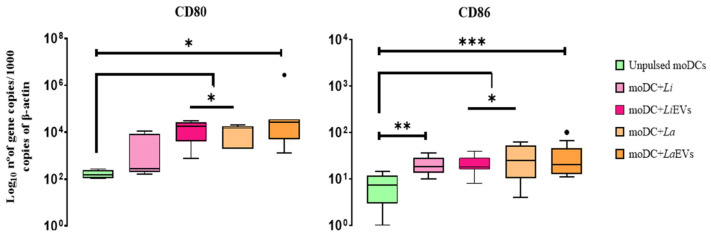
Gene expression of co-stimulatory molecules in *L. infantum*- and *L. amazonensis*-infected moDCs. RNA extracted from infected moDCs (moDC + Li and moDC + La) and moDCs exposed to parasite EVs (moDCs + LiEVs and moDCs + LaEVs) for 24 h were used to evaluate the gene expression of *CD80* and *CD86.* In parallel, unpulsed moDCs were also assessed. The results of at least seven samples are represented by box plots, including the median, interquartile ranges, and minimum and maximum values. The black dots indicate outliers. Nonparametric Wilcoxon’s test was used for statistical comparisons. * (*p* < 0.05), ** (*p* < 0.01), and *** (*p* < 0.001) indicate significant differences.

Interestingly, EVs of both species of *Leishmania* directed the upregulation of *CD80* and *CD86* (*p* _La and Li CD80_ = 0.0156, *p* _La CD86_ = 0.0117, *p*_Li CD86_ = 0.001). However, only *L. amazonensis* parasites (*p*_CD80_ = 0.0156, *p*_CD86_ = 0.0195) stimulate moDCs to increase the gene expression of both co-stimulatory molecules (Figure 9). On the other hand, *L. infantum*-infected moDCs exhibited significant accumulation of CD86 mRNA (*p* = 0.002) when compared with unpulsed moDCs.

Taken together, these results suggest that EVs shed by *L. infantum* and *L. amazonensis* parasites can lead moDCs to present parasite antigens to CD8^+^ T cells through MHCI. Furthermore, *CD80* and *CD86* upregulation mediates the initial steps of T-cell activation. *L. infantum* parasites can modulate moDCs to present antigens through MHCII. However, these parasites only favor CD86 expression, indicating that infected moDCs may present antigens to CD4^+^ T cells. On the other hand, *L. amazonensis* parasites can stimulate moDCs to express CD80 and CD86 co-stimulatory molecules without enhancing the expression of MHC molecules.

### 3.6. L. infantum and L. amazonensis Trigger moDCs to Release the Chemokine CXCL16

DCs release cytokines, promoting the migration of nearby leukocytes. Chemokines interact with the chemotactic receptor of leukocytes, inducing migration. To examine the effect of *L. infantum* and *L. amazonensis* promastigotes and parasite EVs on chemokine generation, the release of soluble CXCL16 and IL-8 gene expression were evaluated in infected moDCs, moDCs exposed to EVs (LiEVs and LaEVs), and unprimed moDCs.

**Figure 10:**
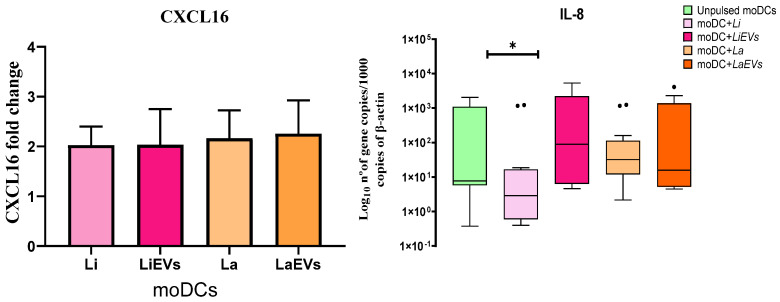
Release of chemokine CXCL16 and gene expression of IL-8 by *L. infantum*- and *L. amazonensis*-infected moDCs. Culture supernatants and RNA extracted from infected moDCs (moDC + Li and moDC + La) and moDCs exposed to EVs (moDCs + LiEVs and moDCs + LaEVs) for 24 h were used to assess soluble CXCL16 and evaluate IL-8 gene expression. The mean and standard deviation of soluble CXCL16 by moDCs are represented by a column bar graph, and IL-8 gene expression is represented by box plots, including the median, interquartile ranges, and minimum and maximum values. The nonparametric Wilcoxon test was used for statistical comparisons. * (*p* < 0.05) indicates statistically significant differences.

Overall, there was at least a two-fold higher concentration of soluble CXCL16 in supernatants of infected moDCs and EV-stimulated moDCs when compared to unprimed moDCs (Figure 10). In turn, *L. infantum*-infected moDCs showed significant downregulation of *IL-8* (*p* = 0.0182).

Therefore, these data indicate that *L. infantum* and *L. amazonensis* parasites, as well as EVs, modulate moDCs to release chemotactic CXCL16, which can promote the migration of immune cells. The induction of CXCL16 release indicates that moDCs are ready to attract T cells, whereas the impairment of IL-8 generation can point to deficient neutrophil recruitment.

### 3.7. L. infantum-Infected moDCs Can Generate IL-1β and IL-18

Depending on the pathogenic stimuli and cytokines that are present in the surrounding microenvironment, DCs can trigger different types of cytokine production. Thus, the gene expression of anti- and pro-inflammatory cytokines was examined in infected and EV-stimulated moDCs, as well as in unprimed moDCs.

**Figure 11:**
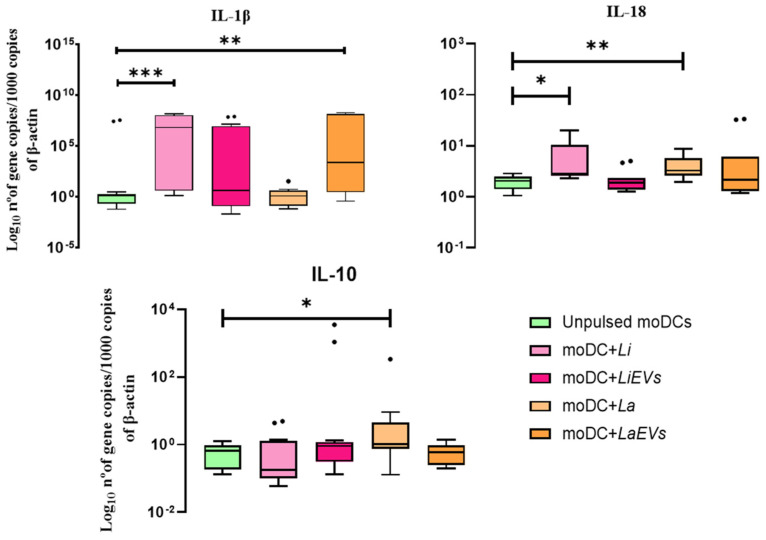
Gene expression of cytokines by *L. infantum*- and *L. amazonensis*-infected moDCs. RNA extracted from infected moDCs (moDC + Li and moDC + La) and moDCs exposed to EVs (moDCs + LiEVs and moDCs + LaEVs) for 24 h were used to evaluate the gene expression of IL-1β, IL-18, and IL-10. In parallel, unpulsed moDCs were also assessed. Cytokine results of at least eight samples are represented by box plots, including the median, interquartile ranges, and minimum and maximum values. The nonparametric Wilcoxon test was used for statistical comparisons. * (*p* < 0.05), ** (*p* < 0.01), and *** *(p* < 0.001) indicate statistically significant differences.

In comparison with unpulsed moDCs, the gene expression of the anti-inflammatory cytokine *IL-10* (Figure 11) was significantly increased in *L. amazonensis*-infected moDCs (*p* = 0.0049) associated with *IL-1α* (*p* = 0.0353) and *TGF-β* downregulation (*p* = 0.0295) (Supplementary Figure S2), whereas moDCs infected with *L. infantum* showed a significant upregulation of *IL-1β* (*p* = 0.007). A significant increase in IL-18 mRNA was also observed in *L. infantum*- (*p* = 0.0391) and *L. amazonensis* (*p* = 0.0049)-infected moDCs. Moreover, the parasites did not seem to affect the gene expression of *IL-12* (*IL-12p35* and *IL-12p40*). However, *L. amazonensis* EVs induced moDCs to significantly upregulate *IL-1β* (*p* = 0.0012), and *L. infantum* EVs promoted the downregulation of *IL-12p40* (*p* = 0.0386).

Thus, in contrast with *L. amazonensis,* which induces moDCs to generate a mix of pro-inflammatory (IL-18) and anti-inflammatory (IL-10) cytokines, *L. infantum* parasites selectively lead moDCs to generate pro-inflammatory cytokines (IL-1β and IL-18) that can be associated with the differentiation of T cells. Furthermore, EVs shed by *L. amazonensis* direct moDCs to generate the pro-inflammatory IL-1β.

## 4. Discussion

The *Leishmania* life cycle is initiated when promastigote forms are introduced into the skin of a mammalian host by female sandflies during blood feeding. Host DCs are recruited at the infection site and take up parasites, increasing the availability of partially processed peptides for presentation via the MHC [45] and acting as inducers of T-cell immune responses [46]. Therefore, the current study examines the immune activation of in vitro differentiated canine moDCs by visceral *L. infantum* and cutaneous *L. amazonensis* promastigotes and by the EVs shed by these parasites.

After seven days in the presence of an rIL-4 and GM-CSF cocktail, monocyte-derived DCs evidence a typical DC-like morphology with long cytoplasmic projections compatible with dendrites. Cytoplasmic projections are the most characteristic morphological features of DCs, which is a distinctive mark when compared with macrophages. However, in addition to their characteristic morphology, dog moDCs evidence a species-specific phenotype. Contrary to human and mouse DCs, canine DCs do not lose the ability to express CD14 after in vitro maturation [37,47,48,49,50]. However, according to previous studies, they can express high levels of MHCII, CD11c [37,46] CD1a, CD40, and CD83, as well as the co-stimulatory molecules CD80 and CD86 [29,50]. Thus, it seems that none of the typical markers commonly used to differentiate DCs in humans and mice, such as CD1c, CD11c, and CD14, are appropriate for the accurate identification of canine DCs. However, in the current study, the moDC population was mainly constituted by CD14^+^, CD11c^+^, CD1a^+^, and CD83^+^ moDC subsets. The moDC population expresses large amounts of CD83^+^, which is a member of the immunoglobulin (Ig) superfamily that predominates in mature DCs, playing a relevant role in the regulation of antigenic presentation. During the in vitro generation of moDCs, IL-4 has been considered essential for the stable expression of surface CD83 [51]. Moreover, in the current study, there was a lower frequency of CD14^+^ moDCs. According to previous studies, these surface markers are present on canine macrophages/monocytes but at lower expression levels when compared to moDCs [28,29,52,53]. moDCs also express CD1a, which facilitates the presentation of non-protein antigens [52] like lipids; CD11, which constitutes the α chain of complement receptor 4 and plays a role in phagocytosis; and CD14, which acts as a coreceptor of TLR4 essential in the recognition of antigenic patterns.

Intracellular *Leishmania* parasites reside in macrophages of the mammalian host but are also phagocytosed by other cells, such as DCs [54]. In addition to being important mediators between innate and adaptive immunity, DCs are also recognized for their highly efficient phagocytic potential [55]. In the current study, dog moDCs phagocytosed both *L. infantum* and *L. amazonensis* promastigotes and supported the differentiation of promastigotes into amastigotes-like forms. These data agree with previous studies on the infection rate of bone marrow-derived dendritic cells (BMDCs) of mice exposed to *L. infantum* [56,57]. For parasite internalization to occur, mobilization of cytoskeletal elements, as well as specific receptor–ligand interactions, have to be established [58]. The parasite gp63 mediates the conversion of complement factor C3b into iC3b, which binds to complement receptor 3, resulting in *Leishmania* adherence to the DC membrane [54]. In human DCs, Argueta-Donohué and colleagues [59] demonstrated that DC-SIGN, a surface receptor primarily found on DCs, efficiently mediates high internalization rates of *L. mexicana* promastigotes after 3 h of in vitro infection. Other in vitro infection studies have collectively indicated that murine mDCs can efficiently engulf promastigotes [54,60,61,62,63]. Margaroni and collaborators [64] showed that almost 50% of BMDCs from mice were effectively infected by *L. infantum* promastigotes after 24 h, demonstrating successful transformation into amastigotes. Moreover, promastigote forms of dermotropic *Leishmania* species, such as *L. major*, *L. amazonensis,* and *L. braziliensis*, resulted in similar infectivity rates, with parasites being able to survive and multiply within BMDCs [57,65,66]. Furthermore, in the current study, the internalized *L. infantum* and *L. amazonensis* parasites appeared to prolong the life span of moDCs, as previously described by Rodrigues and colleagues concerning the lifetime of macrophages infected by *L. infantum* parasites [67].

DCs recognize foreign pathogens through innate receptors, such as TLR and NOD, signalizing downstream pathways that trigger the production of pro-inflammatory cytokines and effector molecules, which promote the differentiation of Th1 cells, leading to an inflammatory immune response. In the current study, short-term exposure to *L. amazonensis* and *L. infantum* EVs modulated moDCs to express *TLR2*, whereas earlier *L. infantum* infection induced moDCs to trigger TLR4 gene expression. On the other hand, *L. amazonensis* parasites do not seem to have any effect on PRR transcription. In addition, the examined endocytic and cytoplasmic PRRs did not appear to be affected by parasites or EVs. In a recent study in murine BMDCs, *L. amazonensis* also appeared to favor the low expression of the PRR adapter MyD88, which is involved in signaling pro-inflammatory pathways [68].

Failure to activate the TLR2 downstream pathway can affect DC activation, influencing the development of the T-cell immune response against *L. infantum* infection [69,70]. On the other hand, in vitro *L. amazonensis* infectivity was much higher in the BMDM of TLR2^−/−^ C57BL/6 mice when compared to wild-type mice. Furthermore, the availability of TLR2 and TLR4 confers macrophage resistance to *L. amazonensis* parasites by inducing the expression of nitric oxide synthase 2, which is involved in the conversion of L-arginine to nitric oxide, leading to parasite death. Therefore, TLR2 deficiency seems to favor polyamine production, which is beneficial for parasite survival and replication [71]. Thus, these findings point out that TLR2 can play a crucial role in the outcome of *Leishmania* infection.

In the current study, *L. infantum* parasites and EVs shed by *L. amazonensis* stimulated the activation of NF-κB, leading to the generation of the pro-inflammatory cytokines IL-1β, as well as IL-18 in the case of *L. infantum*-infected moDCs. In the nucleus, NF-κB acts as a central regulator of pro-inflammatory immune response by inducing the production of chemokines (e.g., CXCL1, CXCL2, and CXCL3) and cytokines (e.g., TNF-α, IL-1β, and IL-6), together with the upregulation of DC co-stimulatory molecules that are essential for T-cell activation. The family of NF-κB transcription factors plays a crucial role in the host’s immune defense against pathogens. Several studies have reported that *Leishmania* and other parasites, such as *Toxoplasma gondii,* interfere with the activation of NF-κB signaling pathways, ensuring their survival in the host [72]. A previous study by Nogueira and collaborators [73] performed with the THP-1 monocyte lineage showed that *L. amazonensis* EVs activate TLR4/TLR2 and induce the nuclear translocation of NF-κB. However, it is difficult to draw a definitive conclusion about the role of NF-κB in *Leishmania* infection, since *Leishmania* parasites appear to induce a pattern of NF-κB-negative modulation. The findings of the current study indicate that *L. amazonensis* EVs shape the activation of NF-κB and the consequent generation of IL-1β by moDCs. Thus, *L. amazonensis* EVs seem to be an attractive tool for the immunomodulation of innate immune response.

It is interesting to note that *L. amazonensis* parasites do not appear to induce the activation and translocation of NF-κB to the nuclei of moDCs. It is widely recognized that parasites have evolved various mechanisms to modulate the host’s immune response. Therefore, *L. amazonensis* parasites may have mechanisms to suppress NF-κB activation, whereas EVs shed by *L. amazonensis* may not have the potential to avoid the activation of NF-κB in recipient cells. In contrast, *L. amazonensis* parasites may use alternative pathways that do not involve NF-κB activation. Although NF-κB is a central regulator of immune responses, there are other signaling cascades, such as MAPK or interferon regulatory factor (IRF) pathways, that may also contribute to the activation of immune cells [66]. *L. amazonensis* can specifically modulate these alternative pathways to manipulate the host’s immune response. MAPK pathways, including extracellular signal-regulated kinase (ERK), c-Jun N-terminal kinase (JNK), and the p38 MAPK pathway, are involved in various cellular processes, including immune responses [74]. Activation of MAPK pathways can lead to positive regulation of CD80 and CD86 expression in DCs. Members of the IRF family, such as IRF3 and IRF7, play a key role in regulating type I interferon (IFN) production and immune response [75]. Activation of the IRF pathways can lead to the production of type I IFNs, such as IFN-α and IFN-β. Type I IFNs, in turn, can induce upregulation of CD80 and CD86 on DCs. The binding of type I IFNs to their receptors on DCs triggers downstream signaling events, including the activation of IRF pathways, resulting in increased expression of CD80 and CD86 [76].

Cytokines play vital roles in immune response toward defense against pathogens. However, the balance of pro- and anti-inflammatory cytokines is crucial to prevent immunopathological disorders. Some of these immune mediators can play a protective role or favor disease progression, while others have a dual role, as is the case of TNF-α and IFN-γ. Although these cytokines play a crucial role in inducing a protective immune response against *Leishmania* infection, their overproduction may have pathological effects, causing severe damage to host tissues [77]. The findings of the current study indicate that *L. infantum* parasites promote the generation of pro-inflammatory cytokines (IL-1β and IL-18) by moDCs, which can favor the development of an inflammatory microenvironment that facilitates the control of *L. infantum* infection. It has been shown that IL-1β can promote the expansion of IL-12-mediated Th1 cells and stimulate NO and TNF production, which may contribute to parasite elimination [78,79]. Mana and coworkers [80] reported that naturally infected, clinically healthy dogs evidence a significant expression of IL-18. In turn, *L. amazonensis*-infected moDCs establish a balance between pro- (IL-18) and anti-inflammatory cytokines (IL-10) that can be favorable to the presence of the parasite. IL-10 is known to be a potent immunoregulatory cytokine, suppressing macrophage activity and DC maturation and inhibiting IL-12 production by DCs, thereby impairing the Th1 immune response and IFN-γ production [81], which limits immune response against intracellular parasitic infection, being associated with the persistence of the parasite at the site of infection [82].

*L. infantum* and *L. amazonensis* EVs drive the expression of MHCI molecules, pointing to the activation of a cytotoxic immune response mediated by T cells, which can promote the apoptosis of infected macrophages. For the initiation of antigen-specific adaptive immune responses, DCs present parasitic antigenic peptides complexed with MHC molecules to naïve T lymphocytes. Parasitic antigens complexed with DC-derived MHCI molecules are cross-presented to CD8^+^ T cells, which become activated into cytotoxic T cells that, in turn, can induce the apoptosis of infected cells. However, the efficient death of infected cells driven by cytotoxic T cells also requires the activation of CD4^+^ T cells via MHCII, which release pro-inflammatory cytokines that strengthen T-cell cytotoxicity. On the other hand, *L. infantum* parasites promote the surface expression of MHCII molecules, pointing out that early moDC infection can favor antigenic presentation, which can induce proliferation and further activation of helper (CD4^+^ T) lymphocytes. Interestingly, *L. amazonensis* parasites do not have a significant effect on the expression of MHCI or MHCII molecules, evading the activation of both cytotoxic and helper T cells.

In the current study, EVs and *L. amazonensis* parasites upregulated both CD80 and CD86 in moDCs, guaranteeing the conditions for the effective activation of T cells following antigen presentation via MHC molecules. In contrast, *L. infantum* parasites only promoted the expression of CD86. According to previous studies, DCs are highly efficient in exogenous antigen cross-presentation to CD8^+^ T cells when compared with macrophages or B cells [83,84,85], since they can efficiently process antigens of pathogens, driving the expression of MHCI and co-stimulatory molecules. Despite the crucial role played by co-stimulatory molecules in T-cell priming, DC surface co-stimulatory molecules CD80 and CD86 bind with low affinity to CD28, which is constitutively expressed by T cells [86]. CD86 is constitutively expressed at low levels in immature DC [87], whereas CD80 is only expressed on activated APCs [88]. Although both CD80 and CD86 are upregulated after DC activation, CD86 expression seems to be induced more rapidly than that of CD80 [89,90]. Neves and coworkers [56] reported that *L. infantum* promastigotes do not increase CD86 expression. However, in the case of human DCs, *L. infantum* and *L. amazonensis* enhance CD86 expression [57]. Although both CD80 and CD86 bind to the same T-cell co-stimulatory molecules to mediate T-cell activation, together with the recognition of parasite antigens complexed with MHC molecules, baseline CD80 could lead to suboptimal T-cell activation.

In the current study, *L. infantum* and *L. amazonensis* parasites, as well as the respective EVs, had a positive effect on the release of soluble CXCL16. The surface expression of CXCL16 on DCs is upregulated by pro-inflammatory cytokines, such as TNF and IFN [91], and the respective receptor (CXCR6) is preferentially expressed on T cells, especially on Th1 lymphocytes, cytotoxic T cells, and memory T cells. It is assumed that CXCL16 attracts T lymphocytes during inflammation, directing T-cell traffic and facilitating immune responses via cell–cell contacts [92,93,94]. Previous studies have reported that *Leishmania* LPG can promote an increase in CXCL16 gene expression via the transcription factor activator protein-1 (AP-1) and NF-κB-dependent transcription [95,96]. The surface of *Leishmania* promastigotes is covered by LPG, and EVs released by promastigotes may also be rich in LPG. Since *L. infantum* EVs and *L. amazonensis* promastigotes did not promote moDCs to increase NF-κB, we can raise the hypothesis that CXCL16 gene transcription is dependent on other transcription factors, as is the case of AP-1.

The findings of the current study indicate that canine DCs differentiated in vitro from peripheral blood monocytes of healthy dogs can recognize parasitic antigens and become activated and may establish a bridge with T cells, favoring the activation of an acquired immune response. However, the activation of moDCs appears to be species-specific. Differences in the activation of skin DCs were also demonstrated in a previous ex vivo study in mice infected with *L. amazonensis* and *L. braziliensis* [97]. *L. infantum* parasites lead to the activation of NF-κB and the subsequent generation of pro-inflammatory cytokines and chemokine release, which can attract T lymphocytes. Moreover, this parasite promotes the expansion of MHCII molecules and the upregulation of CD86, which is described as being expressed earlier and recognized as the initial co-stimulatory molecule. Thus, *L. infantum*-infected moDCs can induce inflammation, drive T-cell chemotaxis, and prime CD4^+^ T cells following parasite antigen presentation, favoring the development of a cellular immune response against infected cells, which can exert some control over infection. On the other hand, *L. amazonensis* promastigotes seem to shape the immune response of moDCs via the activation of other pathways. Since infected moDCs produce CXCL16 and generate pro-inflammatory cytokines, this parasite may signalize moDCs through different pathways without inducing the activation of NF-κB and promote the upregulation of co-stimulatory molecules CD80 and CD86. However, the mixed generation of anti-inflammatory (IL-10) and pro-inflammatory (IL-18) immune mediators can negatively interfere with the activation of the cellular immune response, favoring parasite infection. These findings indicate that *L. amazonensis* and *L. infantum* parasites establish different immune relationships with moDCs, which may be a consequence of a long parasite–dog co-evolution. Indeed, previous studies also point to different strategies adopted by different *Leishmania* species when interacting with DCs, making it very difficult to generalize the effect of this parasite in DCs from different hosts. However, experimental evidence of murine and human DCs has suggested that *L. amazonensis* parasites can impair T-cell activity [98,99,100], which seems to be consistent with the findings of the current study.

*L. amazonensis* and *L. infantum* EVs shape the immune activity of moDCs in different ways. Despite both EVs promoting the release of soluble CXCL16, only *L. amazonensis* EVs induce the activation of NF-κB and the generation of pro-inflammatory immune mediator IL-1β. Nevertheless, both EVs favor MHCI expression in addition to the upregulation of co-stimulatory molecules and CXCL16 release, which points to the stimulation of a cytotoxic immune response mediated by CD8^+^ T cells. Thus, *Leishmania* EVs seem to have the potential to be further developed as an immune modulator of the cytotoxic acquired immune response.

## 5. Conclusions

This study establishes, for the first time, an in vitro dog model to explore the immunomodulation of DCs by *Leishmania*, which may have further applications and open new avenues to innovative DC-based strategies against leishmaniasis.

**Figure 12:**
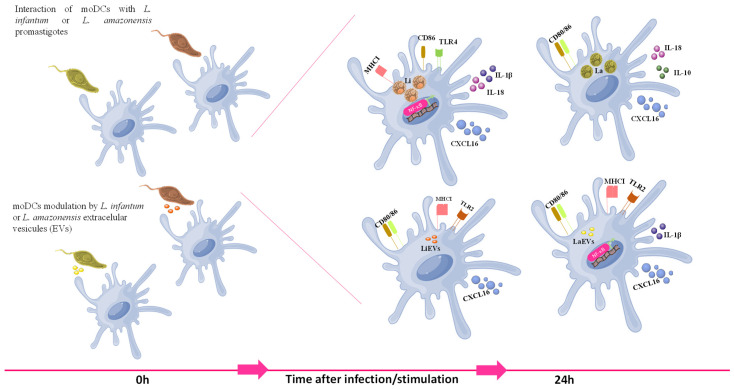
Schematic model reflecting the in vitro interplay established between dendritic cells and *L. infantum*, *L. amazonensis,* and EVs in the early phase of infection. Promastigotes and EVs shed by *L. infantum* (Li) and *L. amazonensis* (La) activate DCs, although in different manners. EVs support the expression of MHCI molecules associated with the expression of co-stimulatory molecules, which indicates that DCs can present parasitic antigens to cytotoxic T cells. The activation and translocation of NF-κB to the DC nucleus seem to be associated with *IL-1β* upregulation. *L. infantum* infection generates pro-inflammatory cytokines (IL-18 and IL-1β) that, together with CXCL16 release, can induce T-cell chemotaxis, facilitating the activation of the acquired immune response. However, in contrast with EV-primed DCs, *L. infantum* infection induces the expression of MHCII but only promotes the upregulation of the co-stimulatory molecule CD86, and *L. amazonensis* upregulates co-stimulatory molecules without interfering with the expression of MHC molecules, which can impact the efficacy of antigenic presentation.

Activated DCs may prevent the development of acute *L. infantum* disease by promoting a cellular immune response that can cause a reduction in infected cells, promoting latent infection due to the low parasite load that favors the development of chronic disease, thereby preventing premature host death. In contrast, *L. amazonensis* parasites appear to impact the immune activation of DCs, which may favour parasite infection. In turn, immune activation of DCs by EVs may lead to the recruitment and activation of cytotoxic T cells, which may exert some control over parasite dissemination (Figure 12).

The co-evolution of *L. infantum* (and eventually *L. amazonensis*) and its animal reservoir has developed a relationship that prolongs both the infection and parasite spread through sandflies. Thus, a detailed understanding of DC activation may offer new opportunities for advanced leishmaniasis control strategies, especially concerning immune-precision therapies. In addition, *Leishmania* EVs have the potential to be exploited as immunomodulators, as they can induce a cytotoxic immune response that could impair host infection.

## Data Availability

Data are available in Zenodo at 10.5281/zenodo.8221227 and upon request from the corresponding author due to privacy.

## Supplementary Materials

The following supporting information can be downloaded at: https://www.mdpi.com/article/10.3390/cells13050445/s1, Table S1. Fluorochrome panel of monoclonal antibodies used to immunophenotype moDCs; Table S2. List of forward (FW) and reverse (RV) primers, base-pair (bp) number of amplified fragments, and primer annealing temperature (TAN) for each studied gene. * indicates the primers designed with Primer3 software 4.1.0; Figure S1. Gene expression of NOD1, NOD2, NLRP10, and TLR9 by *L. infantum*- and *L. amazonensis*-infected moDCs. RNA extracted from infected moDCs (moDC + Li and moDC + La) and moDCs exposed to parasite EVs (moDCs + LiEVs and moDCs + LaEVs) for 24 h were used to evaluate NOD1, NOD2, NLRP10, and TLR9 gene expression. Results of at least eight samples are represented by box plots, including the median, interquartile ranges, and minimum and maximum values. Black dots are indicative of outliers. Nonparametric Wilcoxon’s test was used for statistical comparisons. * (*p* < 0.05) indicates statistically significant differences; Figure S2. Gene expression of cytokines by *L. infantum*- and *L. amazonensis*-infected moDCs. RNA extracted from infected moDCs (moDC + Li and moDC + La) and moDCs exposed to (moDCs + LiEVs and moDCs + LaEVs) for 24 h were used to evaluate *IL-12p40*, *IL-12p35*, *IL-1α*, and *TGF-β* gene expression. In parallel, cytokines of unprimed moDCs were also assessed. The results of at least 10 samples are represented by box plots, including the median, interquartile ranges, and minimum and maximum values. Black dots are indicative of outliers. Nonparametric Wilcoxon’s test was used for statistical comparisons. * (*p* < 0.05) indicates significant differences. References [32,33,34,35,36,37,38,39,40] are cited in the supplementary materials.
