# Supplementary material for: Exploiting Leishmania—Primed Dendritic Cells as Potential Immunomodulators of Canine Immune Response
Source: Cells. 2024 Mar 3;13(5):445. doi: 10.3390/cells13050445 (PMC10931270; doi:10.3390/cells13050445)
Supplement: Supplementary file 1 [file cells-13-00445-s001.zip › cells-2836094-SI.pdf]

Table S1. Fluorochrome panel of monoclonal antibodies used to immunophenotype moDCs.

**Laser**	**488 nm**				**640 nm**	
**Emission filters**	525/40	585/42	525/40	585/42	665/20	660/20
**Fluorochrome**	FITC	PE	FITC	PE	APC	Alexa Fluor® 647
**Relative Brightness**	3	5	3	5	4	4
**Biomarker**	CD14	CD83	MHC II	HLA ABC	CD11c	CD1a
**Monoclonal antibody**	Mouse anti-Human CD14	Mouse anti-Human CD83	Rat anti-Dog MHC Class II	Mouse anti-Human HLA ABC	Mouse anti-Human CD11c	Mouse anti-Human CD1a
**Clone**	TuK4	HB15e	YKIX334.2	W6/32	BU15	NA1/34-HLK
**Company**	ThermoFisher Scientific	Invitrogen	Bio-Rad	Bio-Rad	ThermoFisher Scientific	Bio-Rad

Table S2. List of forward (FW) and reverse (RV) primers, base-pair number (bp) of amplified fragments, and primer annealing temperature (TAN) for each gene studied. * indicates the primers designed with Primer3 software.

Sauter S.N.; Allenspach K.; Gaschen F.; Gröne A.; Ontsouka E.; Blum J.W. Cytokine expression in an *ex vivo* culture system of duodenal samples from dogs with chronic enteropathies: modulation by probiotic bacteria. *Domest Anim Endocrinol.* 2005, 29, 605-622. doi: 10.1016/j.domaniend.2005.04.006

	Primer	Reference	Fragment size (bp)	TA (ºC)
β-Actin	FW-5′ACGGAGCGTGGCTACAGC3′<br>RW-5′TCCTTGATGTCACGCACGA3′	Sauter et al., 2005	61	60.5
TLR2	FW-5′AATCCCCCGTTCAAGTTGTG3′<br>RW-5′ATGGTTTTGCGGCTCTTCTC 3′	Ishii et al.,2006	101	61
TLR4	FW-5′AGAGGATTTCCCCATTGGAC3′<br>RW-5′ACGCAGGTAGCTTGAAGGAA3′	*	86	56
TLR9	FW-5′ACCACATCATCACCCTGGCACCT3′<br>RW-5′ CGGCGACAGTTCCCACCCAC 3′	Rodrigues et al., 2017	82	64
NOD1	FW-5′ CCTTGGCTGTCGGAGATTGGCT 3′<br>RW-5′ ACCTGCTTACTGGGTCGGTGT 3′	Rodrigues et al., 2017	82	61
NOD2	FW-5′ TGGCGTGGGAGCAGGGTTTC 3′<br>RW-5′ CGCTGGGAGGATGTGAAGATGG 3′	Rodrigues et al., 2017	76	66
NLRP10	FW-5′CCATGAGTGTGCGTGGATAC3′<br>RW-5′TGTGCAAGGGTGTGTTTCAT3′	*	73	57
CD80	FW-5′GCAGCAGAAGCCATGGATTAC3′<br>RW-5′CACCAAGAGCTGAGAGACCTTGA3′	Yasunaga et al., 2003	84	60
CD86	FW-5′CGAAACCCACCCCTGATG3′<br>RV-5′CACAAAATGACCAACATTACAAGCA3′	Yasunaga et al., 2003	70	60
TGF-β	FW-5′CAGAATGGCTGTCCTTTGATGTC3′<br>RW-5′AGGCGAAAGCCCTCGACTT3′	Huang, 2008	79	60
IL-10	FW-5′CAAGCCCTGTCGGAGATGAT3′<br>RW-5′CTTGATGTCTGGGTCGTGGTT3′	Yu et al., 2010	78	54
IL-12p35	FW-5′ATGACGGTCCTGTGCCTTAG3′<br>RW-5′CTGCCTCTTGGGATCCATTA3′	*	102	57
IL-12p40	FW-5′CAGCAGAGAGGGTCAGAGTGG3′<br>RW-5′ACGACCTCGATGGGTAGGC3′	Peters et al., 2005	109	58
IL-1α	FW-5′GTTGTTCATTGCCACACAGG3′<br>RW-5′CAAGGCTGGGTTTCCAGTAA3′	*	93	56
IL-1β	Fw-5′CCATGAGTGTGCGTGGATAC3′<br>Rv-5′TGTGCAAGGGTGTGTTTCAT3′	*	80	60.5
IL-8	FW-5′CACTCCACACCTTTCCATCC3′<br>RW-5′GTCCAGGCACACCTCATTTC3′	Harman et al., 2014	120	60
IL-18	FW-5′CTCTCCTGTAAGAACAAAACTATTTCCTT3′<br>RW-5′GAACACTTCTCTGAAAGAATATGATGTCA3′	Argyle et al., 1999	100	56

Ishii M.; Hashimoto M.; Oguma K.; Kano R.; Moritomo T.; Hasegawa A. Molecular cloning and tissue expression of canine Toll-like receptor 2 (TLR2). *Vet Immunol Immunopathol.* **2006**, 110, 87-95. doi: 10.1016/j.vetimm.2005.09.007.

Rodrigues A.; Claro M.; Alexandre-Pires G.; Santos-Mateus D.; Martins C.; Valério-Bolas A.; Rafael-Fernandes M.; Pereira M.A.; Pereira da Fonseca I.; Tomás A.M; Santos-Gomes G. *Leishmania infantum* antigens modulate memory cell subsets of liver resident T lymphocytes. *Immunobiology* **2017**, 222, 409-422. doi: 10.1016/j.imbio.2016.08.009.

Yasunaga S.; Masuda K.; Ohno K.; Tsujimoto H. Antigen-specific enhancements of CD80 mRNA expression in experimentally sensitized dogs with Japanese cedar pollen. *J Vet Med Sci.* **2003**, 65, 295-300. doi: 10.1292/jvms.65.295

Huang Y.C.; Hung S.W-; Jan T.R.; Liao KW.; Cheng C.H.; Wang Y.S.; Chu R.M. CD5-low expression lymphocytes in canine peripheral blood show characteristics of natural killer cells. *J Leukoc Biol.* **2008**, 84,1501-1510. doi: 10.1189/jlb.0408255

Yu D.H.; Noh D.H.; Song R.H.; Park J. Ethyl pyruvate downregulates tumor necrosis factor alpha and interleukin (IL)-6 and upregulates IL-10 in lipopolysaccharide-stimulated canine peripheral blood mononuclear cells. *J Vet Med Sci.* **2010**, 72, 1379–1381. doi: 10.1292/jvms.09-0590

Peters I.R.; Helps C.R.; Calvert E.L.; Hall E.J.; Day M.J. Cytokine mRNA quantification in histologically normal canine duodenal mucosa by real-time RT-PCR. *Vet Immunol Immunopathol***.** **2005**, 103, 101-111. doi: 10.1016/j.vetimm.2004.08.020

Harman R.M.; Bussche L.; Ledbetter E.C.; Van de Walle G.R. Establishment and characterization of an air-liquid canine corneal organ culture model to study acute herpes keratitis. *J Virol.* **2014**, 88, 13669–13677. https://doi.org/10.1128/JVI.02135-14

Argyle D.J.; McGillivery C.; Nicolson L.; Onions D.E. Cloning, sequencing, and characterization of dog interleukin-18. *Immunogenetics* **1999**, 49, 541-543. doi: 10.1007/s002510050532.

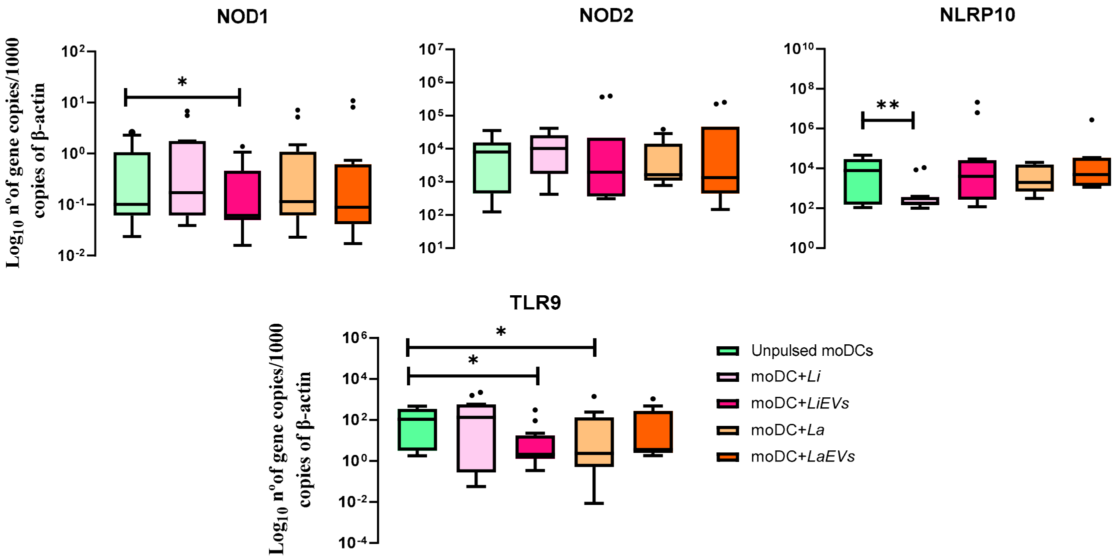

Figure S1. Gene expression of NOD1, NOD2, NLRP10, and TLR9 by *L. infantum* and *L. amazonensis* infected moDCs. RNA extracted from infected moDCs (moDC+*Li* and moDC+*La*) and moDCs exposed to parasite EVs (moDCs+LiEVs and moDCs+LaEVs) for 24 hours were used to evaluate NOD1, NOD2, NLRP10, and TLR9 gene expression. Results of at least eight samples are represented by box plots, including the median, interquartile ranges, and minimum and maximum values. Black dots are indicative of outliers. Nonparametric Wilcoxon's test was used for statistical comparisons. * (p<0.05) indicates statistically significant differences.

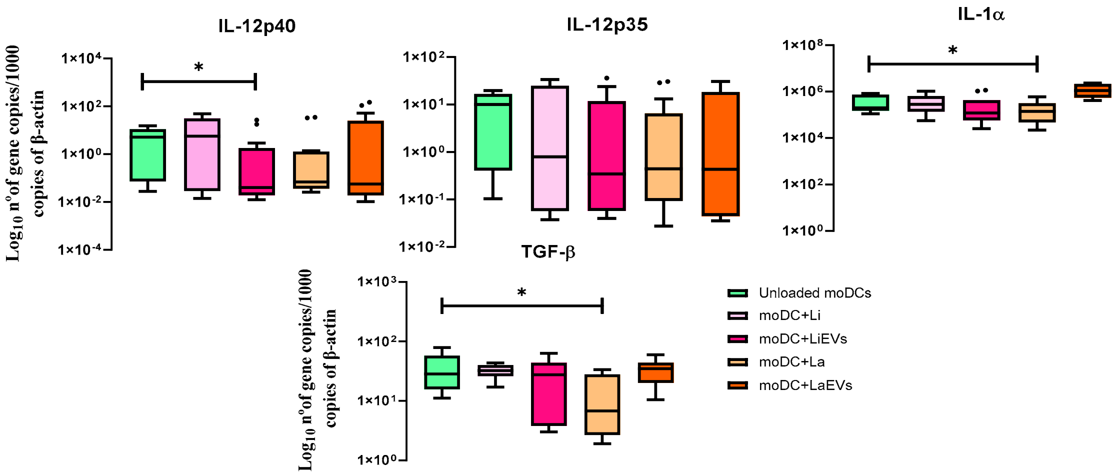

Figure S2. Gene expression of cytokines by *L. infantum* and *L. amazonensis* infected moDCs. RNA extracted from infected moDCs (moDC+Li and moDC+La) and moDCs exposed to (moDCs+LiEVs and moDCs+LaEVs) for 24 hours were used to evaluate *IL-12p40*, *IL-12p35*, *IL-1a*, and *TGF-b* gene expression. In parallel, cytokines of unloaded moDCs were also assessed. The results of at least 10 samples are represented by box plots, including the median, interquartile ranges, and minimum and maximum values. Black dots are indicative of outliers. Nonparametric Wilcoxon's test was used for statistical comparisons. *(p<0.05) indicates significant differences.

.
